# Facile Obtainment of Fluorescent PEG Hydrogels Bearing Pyrene Groups by Frontal Polymerization

**DOI:** 10.3390/polym15071687

**Published:** 2023-03-28

**Authors:** Ricardo D. Martínez-Serrano, Fabián Cuétara-Guadarrama, Mireille Vonlanthen, Javier Illescas, Xiao-Xia Zhu, Ernesto Rivera

**Affiliations:** 1 Instituto de Investigaciones en Materiales, Universidad Nacional Autónoma de México, Circuito Exterior, Ciudad Universitaria, Mexico City CP 04510, Mexico; ricard-d@hotmail.com (R.D.M.-S.); fabian.cuetara@comunidad.unam.mx (F.C.-G.); mireille.vonlanthen@gmail.com (M.V.); 2 Tecnológico Nacional de México/Instituto Tecnológico de Toluca, Avenida Tecnológico S/N Col. Agrícola Bellavista, Metepec CP 52149, Mexico; fillescasm@toluca.tecnm.mx; 3 Département de Chimie, Université de Montréal, Succursale Centre-Ville, Montréal, QC H3C 3J7, Canada; julian.zhu@umontreal.ca

**Keywords:** pyrene, frontal polymerization, hydrogel, fluorescence

## Abstract

Frontal polymerization (FP) was used to prepare poly(ethylene glycol) methyl ether acrylate (PEGMA) fluorescent polymer hydrogels containing pyrenebutyl pendant groups as fluorescent probes. The polymerization procedure was carried out under solvent-free conditions, with different molar quantities of pyrenebutyl methyl ether methacrylate (PybuMA) and PEGMA, in the presence of tricaprylmethylammonium (Aliquat 336^®^) persulfate as a radical initiator. The obtained PEGPy hydrogels were characterized by FT-IR spectroscopy, confirming the effective incorporation of the PybuMA monomer into the polymer backbone. The thermal properties of the hydrogels were determined using thermogravimetric analysis (TGA) and differential scanning calorimetry (DSC). After immersing the hydrogels in deionized water at 25 °C and pH = 7, their swelling behavior was investigated by mass gain at different pH and temperature values. The introduction of PybuMA comonomer into the hydrogel resulted in a decreased swelling ability due to the hydrophobicity of PybuMA. The optical properties of PEGPy were determined by UV-visible absorption and fluorescence spectroscopies. Both monomer and excimer emission bands were observed at 379–397 and 486 nm, respectively, and the fluorescence spectra of the PEGPy hydrogel series were recorded in different solvents to explore the coexistence of monomer and excimer emissions.

## 1. Introduction

Hydrogels are three-dimensional polymeric materials composed of a crosslinked network of hydrophilic chains that possess a porous network, enabling them to absorb and retain large amounts of liquid, primarily in aqueous solutions such as physiological fluids [1,2]. Owing to their high water content and soft and elastic structure, hydrogels display remarkable biocompatibility and biodegradability, making them ideal candidates for a variety of biomedical and pharmaceutical applications, including 3D cell culture, tissue engineering, bioimaging, drug delivery, photodynamic therapy (PDT), wearable diagnosis, biosensing, and environmental remediation [3,4,5,6,7,8,9]. Furthermore, smart hydrogels are sophisticated materials that respond to external stimuli, such as pH, temperature, ionic strength, and solvent polarity, by changing their swelling behavior [10,11]. Fluorescent hydrogels with unique features can be formed by incorporating fluorophores into their reticular network, including π-conjugated organic molecules (pyrene, coumarin, porphyrin, oligothiophene, BODIPY), aggregation-induced emissive dyes (tetraphenylene), lanthanide-doped nanoparticles, carbon dots, and proteins (green fluorescent proteins) [12,13,14]. The resulting fluorescent polymers have found applications in optical device development [15,16] and bioimaging [17,18].

Pyrene, a well-studied organic fluorophore, is a polycyclic aromatic hydrocarbon notable for exhibiting a high extinction coefficient, long fluorescence lifetime, high quantum yield, and facile excimer formation [19]. Upon the local excitation of a ground-state pyrene unit, monomer emission occurs in the region between 375 and 410 nm [19]. Dynamic excimer emission arises following diffusion-controlled collisions between an excited state pyrene unit and a ground-state neighboring pyrene. The excimer emission band of the pyrene excimer appears at a longer wavelength, around 480 nm. Pyrene excimer emission has been used as a fluorescent probe to investigate the local volume and internal dynamics (ID) of polymer chains and dendrimers in solution [20,21,22]. The coexistence of monomer and excimer emission bands has allowed investigations into numerous applications in organic electronic devices [23,24], protein conformation [25], and chemosensing [26]. Pyrene fluorophore has been included as a comonomer in polymeric materials to yield photoluminescent thermosensitive N-isopropyl acrylamide (NIPAAm) hydrogels [27], multi-responsive starPEG hydrogels [28], conductive self-healing hydrogel composites [29], and fluorescent polymeric chemosensors [30].

Frontal polymerization (FP) is a synthetic procedure that involves the application of energy, in the form of heat or UV light, to a localized section of the reaction vessel to initiate a propagating polymerization front [31,32,33]. This front is self-sustained and propagates in a directional fashion along a mixture of monomers and initiators in a manner analogous to a reaction wave, until the monomers have been converted into a polymeric material [34,35]. One of the main advantages of FP over traditional batch polymerization techniques is its high conversion efficiency, with conversions exceeding 99% yield. Additionally, FP is relatively easy to set up, with simple, easy-to-follow protocols and short reaction times of just a few minutes. The energy requirements of FP are also relatively low, with only the necessary energy required to form and sustain the propagating front. Furthermore, FP can be performed under solvent-free polymerization conditions [36]. The features mentioned above make FP an environmentally friendly and energy-efficient process. 

FP has been used in the production of a variety of different polymeric and composite materials, including poly(N-vinylpyrrolidone) under solvent-free conditions, diurethane diacrylates, semi-interpenetrating polymer networks of methyl cellulose, polyacrylamide, and patterned gels by microfluidic-assisted FP [31,37]. Hydrogels have also been prepared using FP, for instance, pH/temperature-responsive hydrogels [38], 2-hydroxyethyl acrylate hydrogels [39], and supramolecular hydrogels of poly(N-isopropylacrylamide) with cyclodextrin [40]. These examples demonstrate that FP has broad applicability and versatility for the preparation of novel polymeric and composite materials.

Our research group has focused on the study of diverse pyrene systems, such as pyrene-labeled dendronized porphyrins [41], donor-acceptor molecules with a pyrene-BODIPY architecture [42], pyrene-porphyrin dyads in flexible dendritic systems, and cyclen-cored star compounds decorated with pyrene and their copper complexes [43]. Furthermore, we have successfully used FP to prepare fluorescent acrylate polymers containing pyrene and hydrogels containing tetraphenylporphyrin fluorophores [44,45,46]. In the present study, we report the facile preparation of a novel series of pyrene-containing poly(ethylene glycol) methyl ether acrylate (PEGMA) fluorescent hydrogels (PEGPy) using FP. The hydrogels were synthesized by heating PEGMA in the presence of tricaprylmethylammonium (Aliquat 336^®^) persulfate (APSO) as a radical initiator, along with varying concentrations of pyrenebutyl methyl ether methacrylate (PybuMA) comonomer. The PEGPy hydrogels were obtained in solvent-free conditions with minimal heat energy input. The thermal and photophysical properties of the PEGPy hydrogels were assessed, and their swelling behavior was determined under different pH and temperature conditions. We have further evaluated the effect of the comonomer concentration on the front velocity (*V*_f_) and maximum temperature (*T*_max_), and the optical properties of the PEGPy hydrogels were recorded after swelling in different organic solvents and water. 

## 2. Materials and Methods

### 2.1. General Notes

All the reagents employed in synthesizing the PybuMA comonomer and PEGPy hydrogels were acquired from Merck Sigma-Aldrich, Toluca, Mexico. Purification of poly(ethylene glycol) methyl ether acrylate (PEGMA, *M_n_* = 480, *d* = 1.09 g mL^–1^, ≥95%) was performed by column chromatography on neutral alumina to eliminate inhibitors before conducting FP experiments. Tricaprylmethylammonium chloride (Aliquat 336^®^, MW = 404.16 g mol^−1^, *d* = 0.88 g mL^−1^, ≥88.2–93.0%) and ammonium persulfate (APS, MW = 228.20 g mol^−1^) were utilized to prepare APSO radical initiator. Moreover, 1-Pyrenebutanol (Pybu), triethylamine (Et_3_N), potassium carbonate (K_2_CO_3_), and methacryloyl chloride were used to synthesize PybuMA comonomer.

### 2.2. Characterization of PybuMA Comonomer and the Obtained PEGPy Hydrogels

Fourier-transform infrared (FT-IR) spectra were acquired on a Thermo Fischer Scientific Nicolet 6700 diamond-based instrument (Waltham, MA, USA) with a Smart Orbit attenuated total reflection (ATR). The ^1^H and ^13^C NMR spectra of the PybuMA comonomer were obtained on a Bruker Advance 400 MHz spectrometer operating at 400 and 100 MHz, respectively. Deuterated chloroform (CDCl_3_) was used as a solvent. A Jeol JMS-T100LC instrument was employed to record direct analysis in real time (DART) mass spectra. Thermal gravimetric analysis (TGA) and dynamic scanning calorimetry (DSC) were employed to investigate the thermal properties of the PEGPy polymer series. TGA was performed using a TGA Q5000IR instrument at a heating rate of 10 °C min^−1^. DSC measurements were obtained on a DSC 2910 TA Instrument by performing two consecutive scans from −90 to 200 °C, employing a heating rate of 5 °C min^−1^ under an inert nitrogen (N_2_) atmosphere. The glass transition temperature (*T*_g_) values were determined from the second thermal scan. UV-vis spectra of the PybuMA fluorophore and the obtained PEGPy hydrogels were acquired using a Shimadzu U-2600 spectrophotometer (Kyoto, Japan), using quartz cell cuvettes of 1.0 cm width. Fluorescence spectra were acquired on a Horiba Fluorolog 3 fluorometer equipped with a xenon lamp and the slits were set to 1 nm for excitation and emission spectra. Spectrophotometric-grade tetrahydrofuran (THF) was used as a solvent.

### 2.3. Synthesis of 4-(Pyren-1-yl)Butyl Methacrylate (PybuMA)

In a round-bottom flask, Pybu (0.40 g, 1.4 mmol) was dissolved in 15 mL of dry dichloromethane under an inert atmosphere, followed by the addition of Et_3_N (1.20 mL, 8.2 mmol). The reaction mixture was left to cool in an ice bath. Afterwards, methacryloyl chloride (0.12 mL, 1.1 mmol) was added dropwise to the reaction mixture, which was then allowed to reach room temperature and left stirring for 48 h. To transform the nonreactive acyl chloride groups into methyl ester, methanol (MeOH) was added. The reaction mixture was washed three times with an aqueous solution of K_2_CO_3_ and the organic phase was dried with sodium sulfate (Na_2_SO_4_) before being concentrated under reduced pressure. The purification of the crude product was performed by silica gel column chromatography, using hexanes/ethyl acetate (AcOEt) 80/20 as eluent. The pure product PybuMA was obtained as a yellow solid (0.45 g) with an overall yield of 96%. ^1^H-NMR (400 MHz, CDCl_3_, δ): 8.26 (d, *J* = 9.1 Hz, 1H, C*H* Ar_py_), 8.16 (d, *J* = 7.5 Hz, 1H, C*H* Ar_py_), 8.15 (d, *J* = 7.5 Hz, 1H, C*H* Ar_py_), 8.10 (d, *J* = 7.7 Hz, 1H, C*H* Ar_py_), 8.08 (d, *J* = 9.3 Hz, 1H, C*H* Ar_py_), 7.86 (d, *J* = 7.8 Hz, 1H, C*H* Ar_py_), 6.10 (dq, *J* = 1.6, 1.0 Hz, 1H, C=CH(*H*)), 5.55 (dq, *J* = 1.6, 1.6 Hz, 1H, C=CH(*H*)), 4.24 (t, *J* = 6.4 Hz, 2H, C*H*_2_O), 3.39 (t, *J* = 7.6 Hz, 2H, C*H*_2_Py), 2.04–1.98 (m, 2H, C*H*_2_), 1.97 (dd, *J* = 1.6, 1.0 Hz, 3H, C*H*_3_), 1.93–1.87 (m, 2H, C*H*_2_). ^13^C-NMR (100 MHz, CDCl_3_, δ): 136.4, 131.4, 130.9, 129.8, 128.6, 127.5, 127.3, 127.2, 126.6, 125.8, 125.3, 125.1, 124.9, 124.8, 124.7, 123.3, 64.5, 33.0, 28.6, 28.1, 18.3 ppm. DART^+^: m/z calculated for C_24_H_22_O_2_ 342.16 [M]^+^; found: 343 (Figures S1–S4).

### 2.4. Frontal Polymerization

The frontal polymerization experiments were performed in 4 cm long and 0.8 cm inner diameter glass test tubes, utilizing a heating iron as the energy source. A mixture of PEGMA monomer (1 mL, 2.3 mmol), PybuMA comonomer, and 4.0 mol% APSO radical initiator was prepared. The monomer solution was thoroughly mixed by vigorous stirring at room temperature. The FP reaction was initiated by locally heating the top level of the solution with the tip of a soldering iron. Once a front was observed, the heating source was withdrawn. The self-sustained hot polymerization front propagated throughout the entire monomer mixture from top to bottom. The maximum temperature of the polymerization front (*T*_max_) was measured using a digital scanning thermometer (Omega HH306A data logger thermometer, Norwalk, CT, USA) with two K-type thermocouples. The thermocouples were located at a distance of 1 cm from the top level of the solution and 1 cm apart from each other, respectively. The front velocity (*V*_f_), expressed in cm min^−1^, was determined by recording the time taken for the front to pass from the first thermocouple to the second. The glass test tubes were carefully broken into pieces and the formed hydrogels were thoroughly cleaned of glass debris. Hydrogels were immersed in a mixture of MeOH:H_2_O (1:1) and left stirring for a duration of 48 h at room temperature to eliminate any non-reactive reactants. Subsequently, the hydrogels were dried via vacuum filtration to remove the washing solution.

### 2.5. Swelling Measurements

The hydrogel samples were sliced into small, uniformly sized disks. The dry disks were weighed (*M_d_*) and immersed in distilled water at 25 °C. The weight of each disk was recorded at various time intervals until no further weight gain was observed, indicating the attainment of swelling equilibrium. The swelling ratio (*SR*%) was calculated as weight gain according to the following equation:

(1) $$SR\%=\frac{M_{s}-M_{d}}{M_{d}}\cdot 100$$

where *M_s_* and *M_d_* represent the sample weight in its swollen and dry states, respectively. 

The pH effect on the swelling behavior of the hydrogels was also investigated. The *SR*% values were determined at different pH values ranging from 2 to 8 after 24 h of immersion at 25 °C in citrate-phosphate buffer solution with the corresponding pH value. Swelling behavior was also evaluated at different temperatures ranging from 20 to 70 °C. The hydrogel disks were immersed in deionized water for 24 h at each temperature and the *SR*% value was determined. 

## 3. Results and Discussion

### 3.1. Synthesis of the PybuMA Monomer

**Scheme 1:**
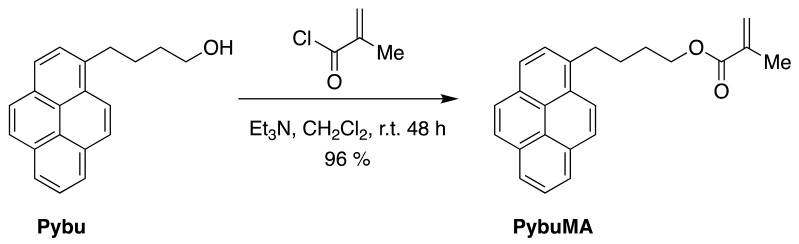
Synthesis of PybuMA monomer.

PybuMA was synthesized in a one-step reaction using a method previously reported by our research group (Scheme 1) [44,45]. For the synthesis, 1-pyrenebutanol (Pybu) was reacted with methacryloyl chloride in CH_2_Cl_2_ at room temperature in the presence of Et_3_N to obtain the desired PybuMA comonomer. The resulting monomer was fully characterized by both NMR spectroscopy and DART mass spectrometry. 

### 3.2. Frontal Polymerization of PEGPy Hydrogel

**Scheme 2:**
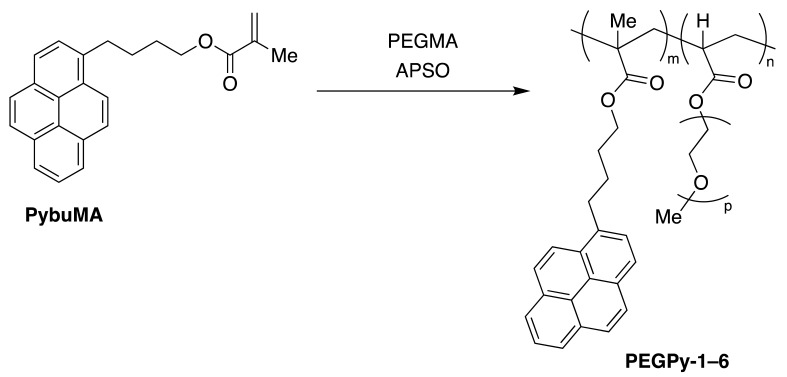
Synthetic scheme for the preparation of the PEGPy-1–6 hydrogel series obtained by FP.

Thermal FP of PEGPy hydrogel series was performed using PEGMA monomer, APSO initiator (4.0 mol%), and PybuMA comonomer in concentrations ranging from 0 to 10 mol% as described in Scheme 2 and Table 1. The *V*_f_ and *T*_max_ values of the self-sustained polymerization front were determined as a function of PybuMA comonomer concentration, and the obtained results are summarized in Table 1.

**Figure 1:**
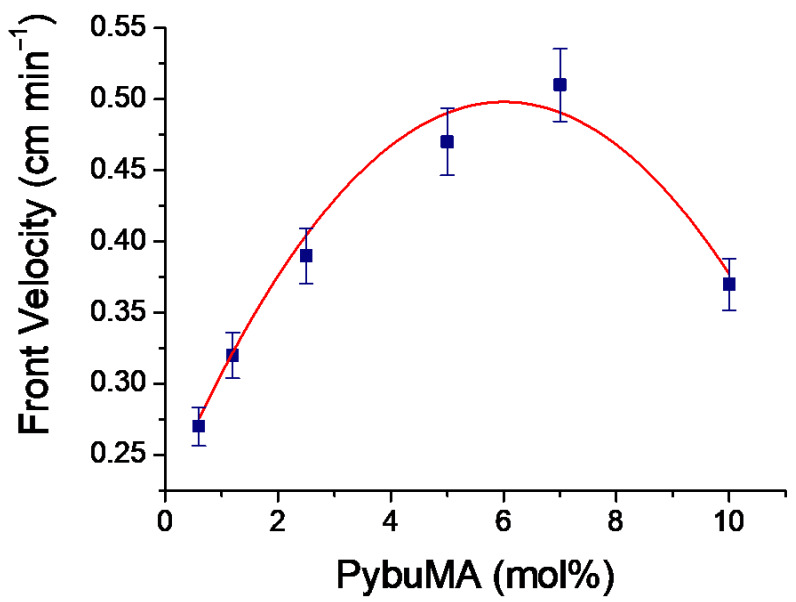
Plot of *V*_f_ vs PybuMA comonomer concentration of the frontal polymerization experiments of PEGPy series.

The effect of the PybuMA comonomer concentration on the *T*_max_ values reached by the polymerization front in the PEGPy series was found to be negligible. However, it was observed that up to 7.0 mol% of PybuMA, the *V*_f_ values increased. Nevertheless, when PybuMA was present in 10 mol%, a significant decrease in *V*_f_ was observed, indicating that the propagating front lost its self-sustaining ability, resulting in an incomplete polymerization process. The above can be rationalized by considering the lower reactivity of PybuMA comonomer with respect to that of PEGMA (Figure 1) [45].

### 3.3. Characterization of the Obtained Hydrogels

**Figure 2:**
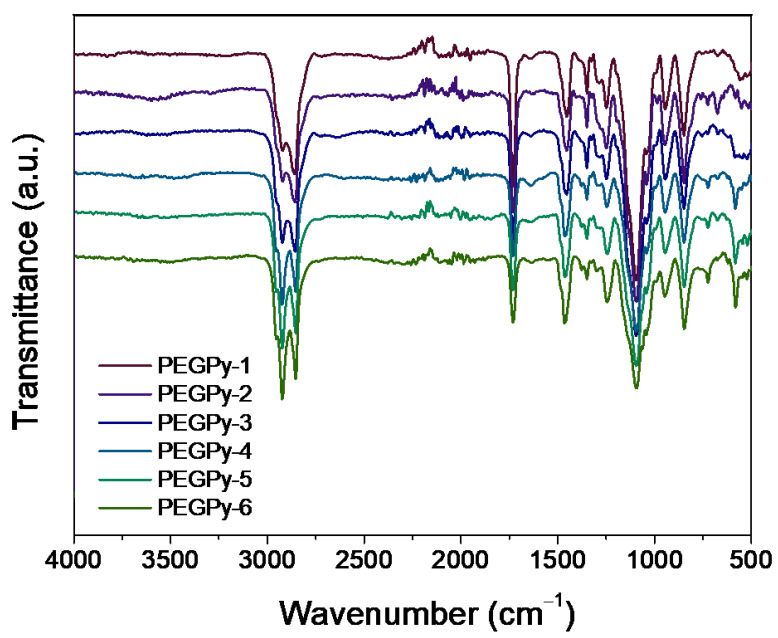
FT-IR spectra of PEGPy hydrogel series.

FT-IR spectra of the PEGPy series exhibited a set of bands corresponding to the functional groups of PybuMA at 2927, 2853 (CH_2_), 1726 (C=O), 1635 (C=C), 1292 (C–O ester), 1087 (C–O ether), and 842 cm^−1^ (=C–H, out of plane). Particularly, the bands at 2927 and 842 cm^−1^ gave evidence of the successful incorporation of the PybuMA comonomer into the polymer backbone (Figure 2).

### 3.4. Thermal Properties of the Obtained Hydrogels

The thermal properties of the PEGPy hydrogel series were determined using TGA and DSC techniques. The reference PEGMA hydrogel without the PybuMA comonomer exhibited a *T*_10_ value of 209 °C and displayed rapid degradation between 326 and 438 °C (Figure S5). The DSC experiment revealed a *T*_g_ value of −61 °C for the reference hydrogel. The PEGPy hydrogel series exhibited *T*_10_ values of approximately 320 °C, with a marked degradation between 346 and 425 °C. Furthermore, the *T*_g_ values of the PEGPy hydrogels were close to −60 °C, which were comparable to that observed for the reference PEGMA hydrogel. These results indicated that the incorporation of the pyrene-containing comonomer PEGPy in the hydrogels did not significantly modify the structure of the bulk polymeric matrix composed of polyethylene glycol. 

### 3.5. Swelling Behavior

**Figure 3:**
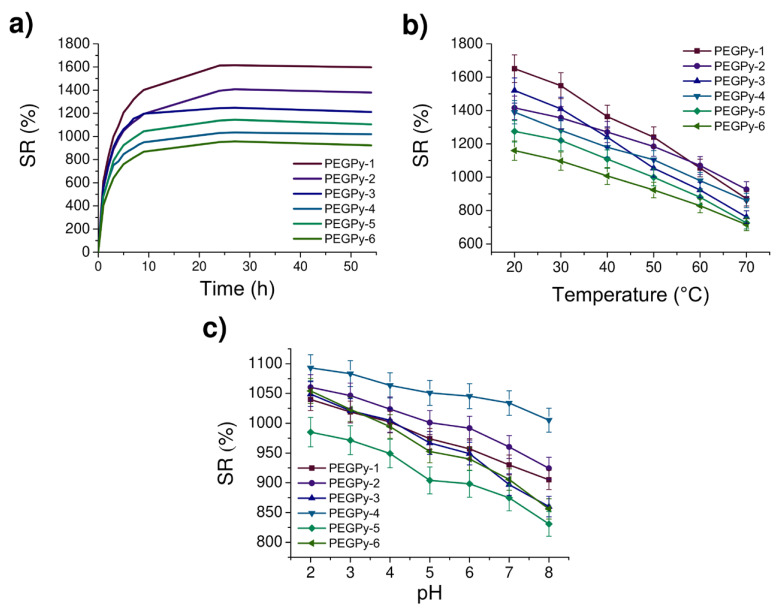
Swelling behavior of the PEGPy hydrogel series under different conditions: (**a**) at pH = 7 and a constant temperature of 25 °C; (**b**) as a function of temperature and; (**c**) as a function of pH change.

The swelling studies of PEGPy hydrogel samples were carried out in deionized water (pH = 7, 25 °C) over a period of 24 h until swelling equilibrium was reached. It was observed that the swelling ratio of PEGPy hydrogels containing higher PybuMA content was reduced, possibly due to the hydrophobic character of pyrene fluorophore units (Figure 3a). At room temperature, the largest swelling ratio value of approximately 1600% was obtained for PEGpy-1 hydrogel, while the lowest, of 900%, was obtained for PEGPy-6. 

The swelling ratio of the PEGPy hydrogel was observed to decrease as the temperature was augmented from 20 to 70 °C (Figure 3b). This finding correlates well with the lower critical solution temperature (LCST) of methacrylate PEG copolymers containing hydrophobic porphyrin derivatives, reported by our research group in a previous study [47]. It is worth mentioning that the swelling ratio values between the PEGPy-1–6 hydrogel series were more distinct at temperatures closer to 20 °C. However, at higher temperatures, around 70 °C, there was less difference in swelling ratio between the series. Furthermore, a pH-dependent swelling response was observed. The hydrogel’s swelling ratio was evaluated at pH values ranging from 2 to 8 using citrate-phosphate buffer solutions at a temperature of 25 °C. It was found that the swelling ratio increased at low pH values (pH = 2) and attained a minimum value at a pH of 8 (Figure 3c).

### 3.6. Optical Properties of PEGPy Hydrogels

**Figure 4:**
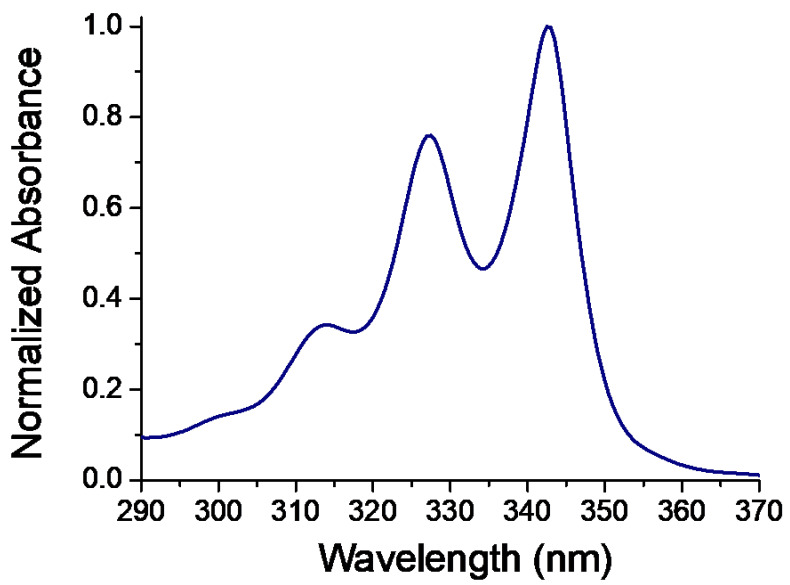
UV-vis absorption spectrum of PybuMA in THF.

The optical properties of the obtained PEGPy hydrogels were studied using absorption and fluorescence spectroscopies. The UV-vis absorption spectrum of PybuMA pyrene comonomer in THF solution featured a prominent absorption band at 345 nm related to the pyrene S_0_–S_2_ transition, as seen in Figure 4.

The fluorescence spectrum of the pyrene moiety displays two distinctive bands, namely, the monomer emission within 360–400 nm and the excimer emission near 480 nm. The interaction between an electronically excited state pyrene molecule and another pyrene in its electronic ground state leads to the formation of a temporary-bound dimer that dissociates upon returning to its ground state. Such a dynamic excimer formation process has previously been reported in the literature [22].

**Figure 5:**
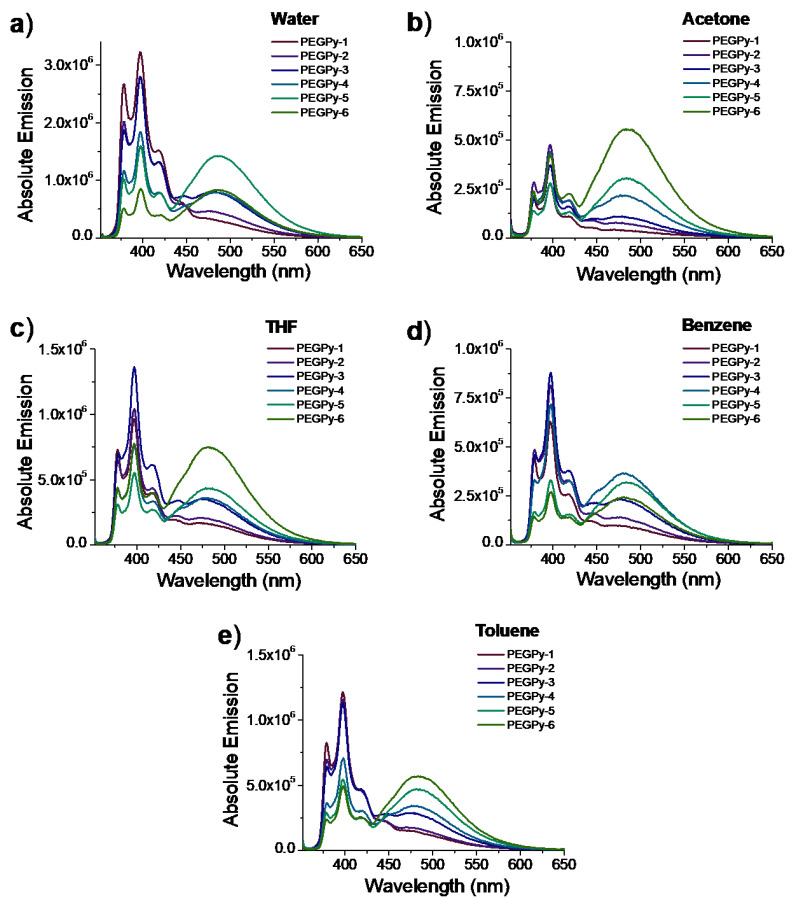
Emission spectra of PEGPy-1–6 hydrogel series in the swollen state in (**a**) water; (**b**) acetone; (**c**) THF; (**d**) benzene and (**e**) toluene.

The solid-state fluorescence spectra of the PEGPy hydrogel series in the swollen state were recorded by exciting at 345 nm and are presented in Figure 5a. The PEGPy hydrogels exhibited both pyrene monomer and excimer emission bands at 379, 397, and 486 nm, respectively. On the one hand, the pyrene monomer emission intensity decreased progressively along the PEGPy series, from 0.6 to 10 mol% of PybuMA comonomer concentration. On the other hand, the excimer emission increased due to the formation of pyrene–pyrene complexes proximal to each other at higher PybuMA concentrations.

It is important to note that in the dry state, hydrogels showed weak emission, which significantly increased upon swelling. This phenomenon can be attributed to the decrease in volume size in the dry state, which promotes the formation of pyrene aggregates and consequently quenches fluorescence. However, in the swollen state, the increase in volume size precludes aggregation, thereby promoting intense fluorescence emission.

The fluorescence spectra of PEGPy hydrogels were recorded in different organic solvents (benzene, toluene, THF, and acetone) and water (Figure 5a–e) to explore the coexistence of pyrene monomer and excimer emissions. In particular, we found that PEGPy-1 and PEGPy-2 exhibited mainly pyrene monomer emission and slight excimer formation in all the solvents explored. With an increase in PybuMA comonomer concentration in PEGPy-3 and PEGPy-4, we observed a significant increase in the excimer emission intensity and a gradual decrease in the intensity of the monomer emission band. Finally, we noted the clear coexistence of pyrene monomer and excimer emission in all the solvents studied for PEGPy-5 and PEGPy-6 emission spectra. 

**Figure 6:**
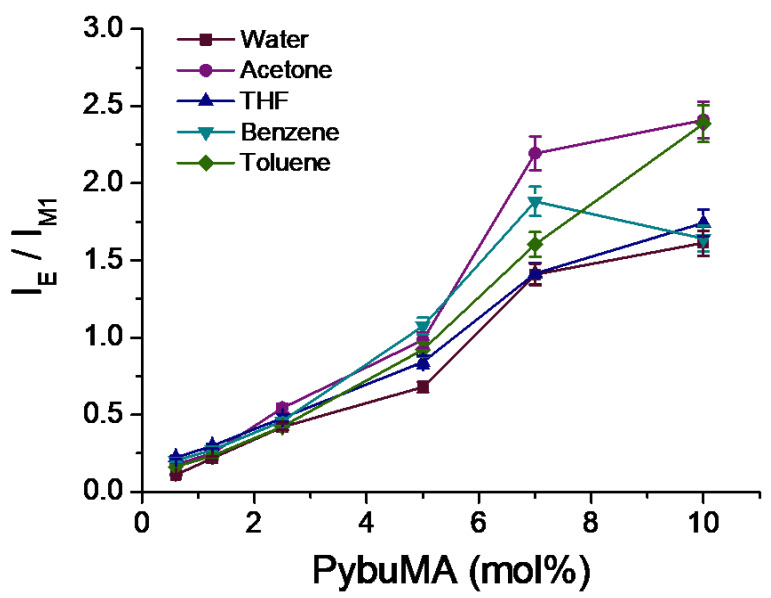
Plot of the I_E_/I_M1_ ratio for the PEGPy-1–6 hydrogels in water, acetone, THF, benzene, and toluene.

To analyze the behavior of the pyrene monomer and excimer bands in organic solvents and water, the intensity ratio between the excimer emission (I_E_ = 486 nm) and the monomer emission (I_M1_ = 379 nm), denoted as I_E_/I_M1,_ was calculated. A plot of I_E_/I_M1_ as a function of PybuMA concentration evidenced the influence of the pyrene content on excimer formation (Figure 6). 

Hydrogels having PybuMA content below 2.5 mol% (PEGPy-3) predominantly presented monomer emission in all studied swelling media. The formation of pyrene excimers was precluded due to the long-distance separation between pyrene units together with the evident steric hindrance produced by PEG sidechains. However, increasing the PybuMA comonomer content within the hydrogel (≥5.0 mol%, PEGPy-4) favored the formation of inner pyrene–pyrene dimers. This evidenced that excimer formation was governed by the enhanced probability of interactions between pyrene groups. Nevertheless, this observation was more pronounced in organic media than in water due to the reduced swelling capacity of PEGPy hydrogels in organic solvents. As a result, pendant pyrene groups are located closer to each other when hydrogels are immersed in organic media rather than aqueous media, resulting in more intense excimer emission.

**Figure 7:**
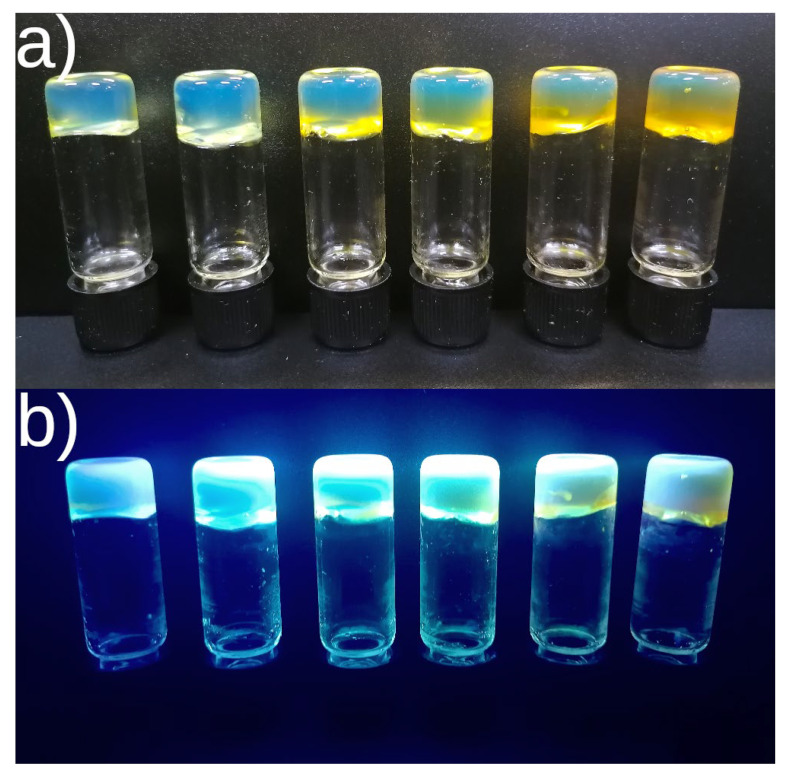
Obtained PEGPy-1–6 hydrogel series (from left to right 0.6 to 10.0 mol%) exposed to: (**a**) visible light and; (**b**) UV light (intense blue color emission can be observed).

The PEGPy hydrogels presented a distinctive yellow coloration under visible light, which was attributed to the PybuMA comonomer (Figure 7a). The characteristic pyrene blue emission was observed when the hydrogels were exposed to UV light (Figure 7b). The observed phenomenon can be explained by the crosslinked structure of the PEGMA matrix, which prevented aggregation-induced quenching by constraining the mobility of the pyrene moieties. 

## 4. Conclusions

A series of pyrene-based hydrogels (PEGPy) with remarkable fluorescent properties were synthesized via FP using APSO as the radical initiator. Frontal polymerization, a facile, simple, and green synthetic methodology, was used to obtain fluorescent PEG acrylate hydrogels. The incorporation of pyrene fluorophore had a negligible impact on the thermal properties of the hydrogels, as evidenced by the comparison of their *T*_10_ and *T*_g_ values to those of the PEGMA polymer matrix. The swelling behavior of the PEGPy hydrogels was influenced by the PybuMA comonomer concentration, which showed a hydrophobic character due to the presence of pyrene moieties. Both the temperature and pH of the swelling medium had a clear influence on the swelling properties, resulting in a decrease in swelling ratio at high temperatures and pH values. Moreover, the obtained hydrogels exhibited the characteristic monomer and excimer emission bands corresponding to a pyrene moiety, with the excimer emission band being intensified for higher PybuMA content. Additionally, the solvent polarity significantly affected the emission properties of the PEGPy hydrogels. The excellent fluorescent, pH-responsive, and thermo-responsive properties of the polymer hydrogels presented in this study make this class of materials promising for various applications such as chemical and environmental sensing, photonics, and bioimaging, among others.

## Figures and Tables

**Table 1:** Content of PybuMA comonomer in PEGPy-1–6 hydrogel series and the *V*_f_ and *T*_max_ parameters of FP.

Sample	PybuMA (mol%)	*V*_f_ (cm min^−1^)	*T*_max_ (°C)
PEGMA	0.0	0.47	95.9
PEGPy-1	0.6	0.27	80.6
PEGPy-2	1.2	0.32	83.9
PEGPy-3	2.5	0.39	87.5
PEGPy-4	5.0	0.47	89.5
PEGPy-5	7.0	0.51	84.1
PEGPy-6	10.0	0.37	86.2

## Data Availability

Data are available within this article and in the associated Supplementary Materials.

## Supplementary Materials

The following supporting information can be downloaded at: https://www.mdpi.com/article/10.3390/polym15071687/s1, Figure S1. Structure of PybuMA, Figure S2. ^1^H-NMR spectrum of monomer PybuMA. Figure S3. ^13^C-NMR spectrum of monomer PybuMA. Figure S4. DART^+^ mass spectrum of monomer PybuMA, Figure S5. TGA curves of the PEGPy polymer series.
