# Supplementary material for: Facile Obtainment of Fluorescent PEG Hydrogels Bearing Pyrene Groups by Frontal Polymerization
Source: Polymers (Basel). 2023 Mar 28;15(7):1687. doi: 10.3390/polym15071687 (PMC10097409; doi:10.3390/polym15071687)
Supplement: Supplementary file 1 [file polymers-15-01687-s001.zip › polymers-2267297-supplementary.pdf]

Supporting information for:

Facile Obtainment of Fluorescent PEG Hydrogels Bearing Pyrene Groups by Frontal Polymerization

Ricardo D. Martínez-Serrano [1], Fabián Cuétara-Guadarrama [1], Mireille Vonlanthen [1], Javier Illescas [2], Xiao-Xia Zhu [3] and Ernesto Rivera [1,*]

[1] Instituto de Investigaciones en Materiales, Universidad Nacional Autónoma de México, Circuito Exterior, Ciudad Universitaria, Mexico City CP 04510, Mexico; ricard-d@hotmail.com (R.D.M.-S.); fabian.cuetara@comunidad.unam.mx (F.C.-G.); mireille.vonlanthen@gmail.com (M.V.)

[2] Tecnológico Nacional de México/Instituto Tecnológico de Toluca, Avenida Tecnológico S/N Col. Agrícola Bellavista, Metepec CP 52149, Mexico; fillescasm@toluca.tecnm.mx

[3] Département de Chimie, Université de Montréal, Succursale Centre-Ville, Montréal, QC H3C 3J7, Canada; julian.zhu@umontreal.ca

* Correspondence: riverage@unam.mx

1. Characterization of the pyrene-containing monomer PybuMA

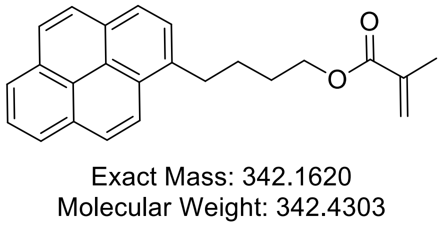

Figure S1. Structure of PybuMA.

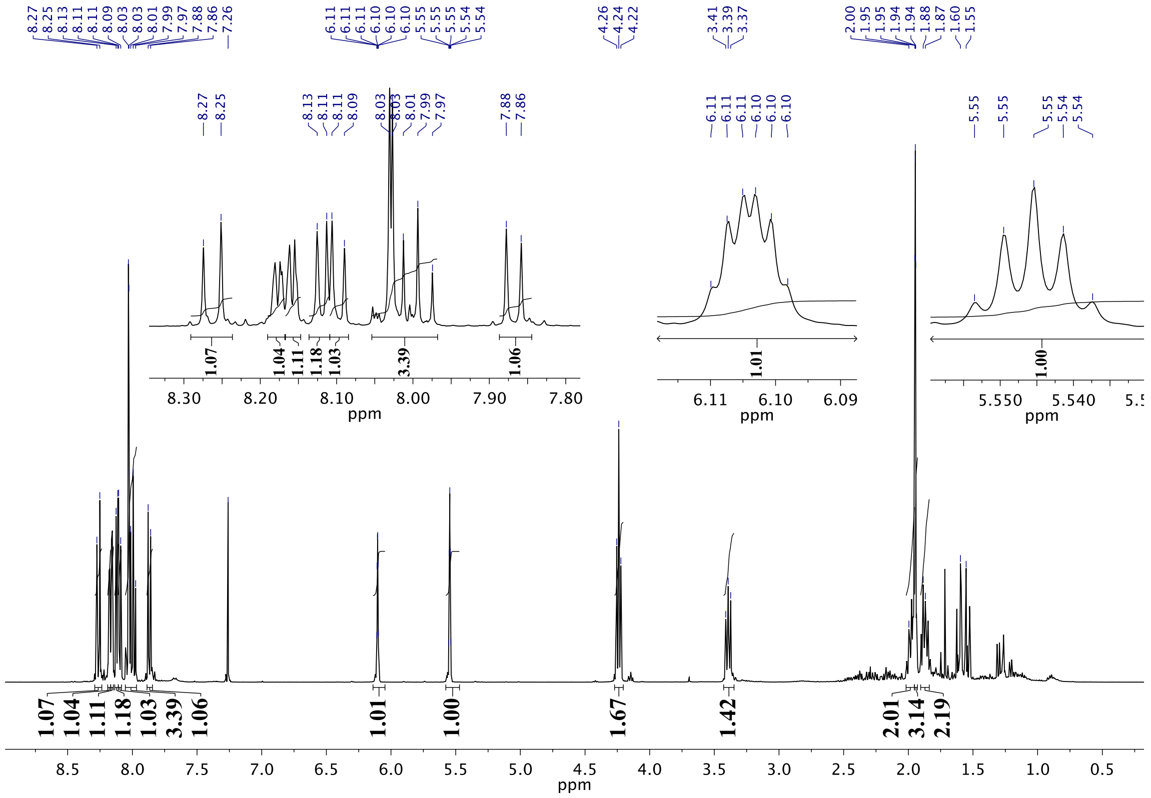

Figure S2. ^{1}H-NMR spectrum of monomer PybuMA.

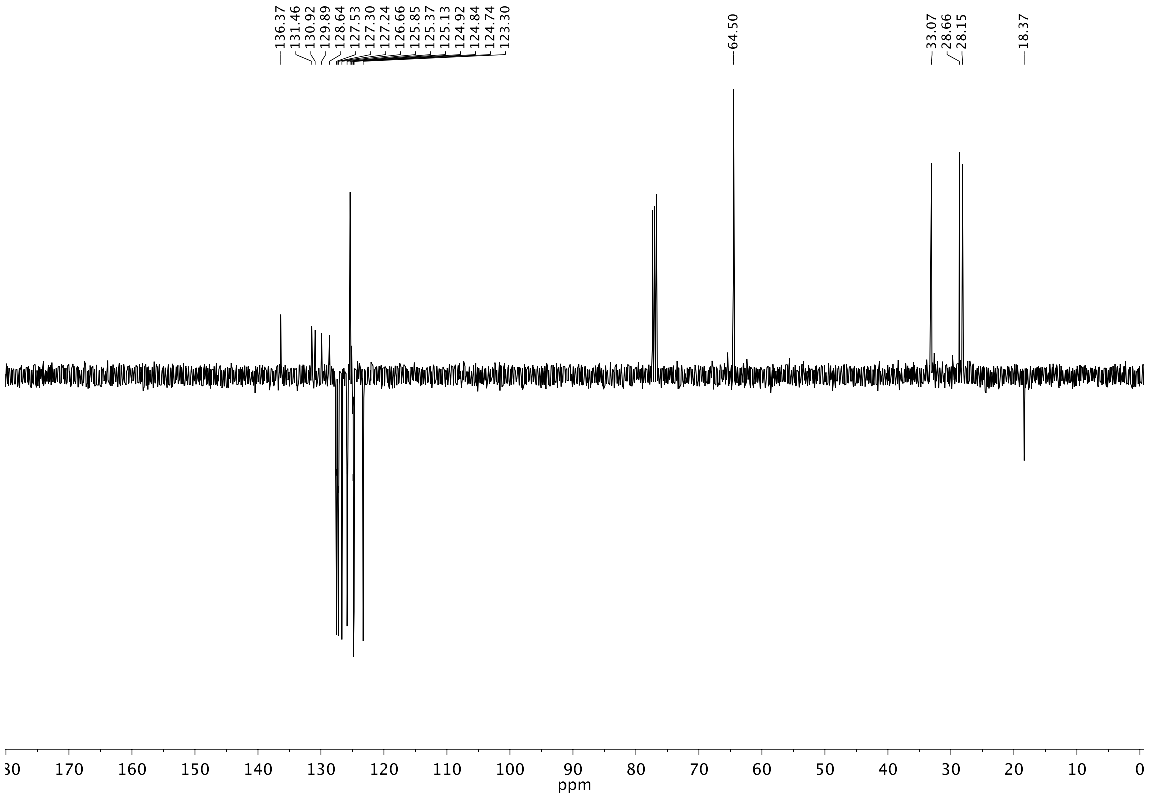

Figure S3. ^{13}C-NMR spectrum of monomer PybuMA.

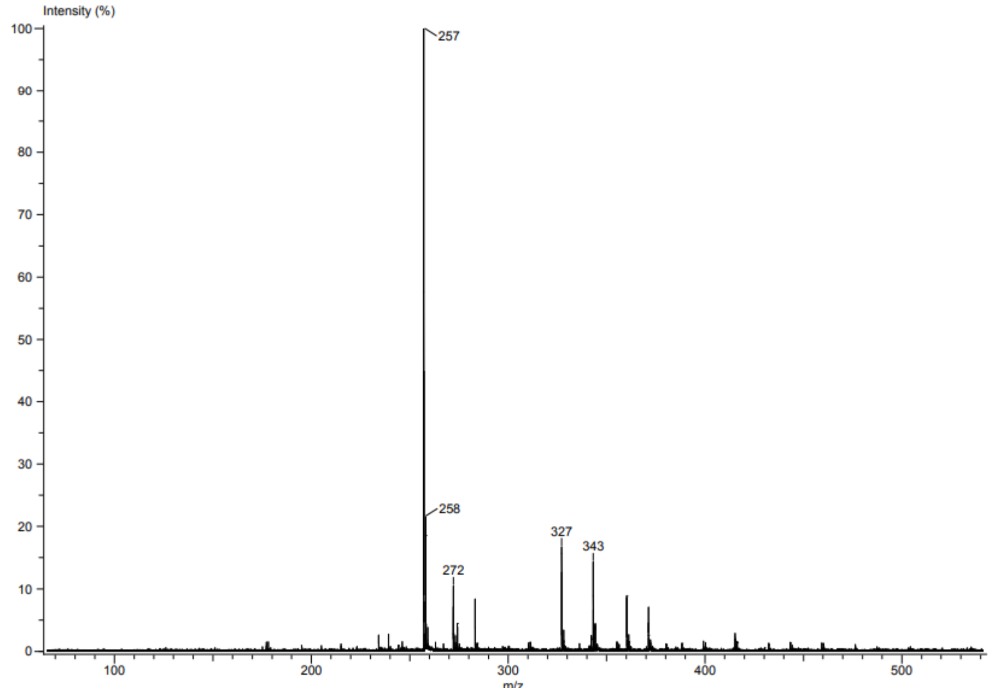

Figure S4. DART^{+} mass spectrum of monomer PybuMA.

2. TGA measurements.

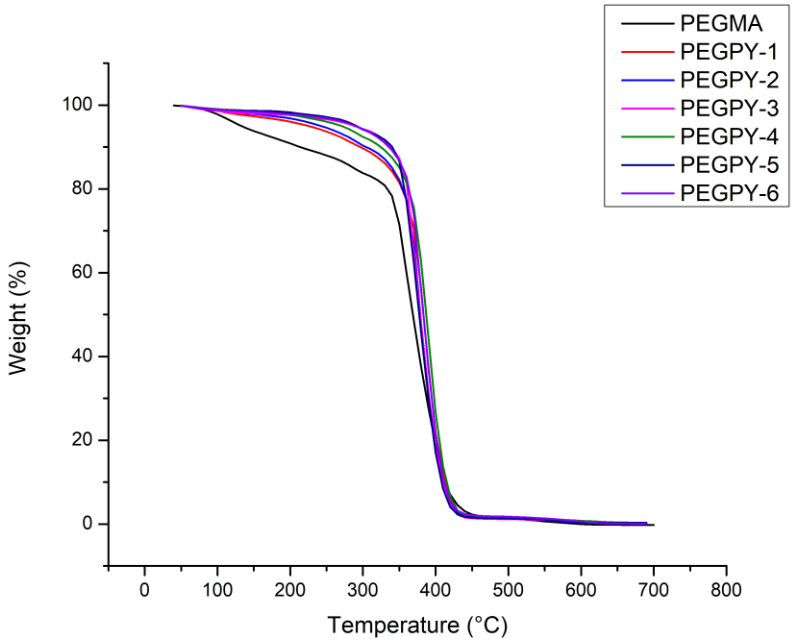

Figure S5. TGA curves of the PEGPy polymer series.
